# Antimicrobial and Flame-Retardant Coatings Prepared from Nano- and Microparticles of Unmodified and Nitrogen-Modified Polyphenols

**DOI:** 10.3390/polym15040992

**Published:** 2023-02-16

**Authors:** Petri Widsten, Satu Salo, Tuula Hakkarainen, Thu Lam Nguyen, Marc Borrega, Olesya Fearon

**Affiliations:** VTT Technical Research Centre of Finland Ltd., Tietotie 2, 02150 Espoo, Finland

**Keywords:** antimicrobial, chemical modification, coating, flame retardant, lignin, microparticles, nanoparticles, tannin

## Abstract

The purpose of this study was to elucidate the structures and functional properties of tannin- and lignin-derived nano- and microparticles and the coatings prepared from them. Nanoparticles prepared from technical lignins and water-insoluble tannin obtained from softwood bark showed large differences in the suspension testing of antibacterial efficacy against methicillin-resistant *Staphylococcus aureus* (MRSA) bacteria. A common factor among the most effective lignin nanoparticles was a relatively low molar mass of the lignin, but that alone did not guarantee high efficacy. Tannin nanoparticles showed good antibacterial activity both in suspension testing and as coatings applied onto cellulose. The nanoparticles of nitrogen-modified tannin and the small microparticles of nitrogen-modified kraft lignin exhibited promising flame-retardant parameters when applied as coatings on cellulose. These results illustrate the potential of nano- and microsized particles of unmodified and chemically modified polyphenols to provide functional coatings to cellulosic substrates for environments and applications with high hygiene and fire safety requirements.

## 1. Introduction

Recent years have seen an emergence of research in functional coatings derived from natural polymers present in lignocellulosic and other biomass [1]. This activity is driven partly by concerns over the impacts on human health and the environment of petroleum-derived chemicals and polymers and partly by the perception that oil supplies may be dwindling. One of the most investigated biopolymers for coating applications is lignin [2,3], a natural polyphenol that is abundantly available as a by-product of lignocellulosic pulping and biorefinery operations. While technical lignins such as kraft lignin (KL), organosolv lignin (OSL), and soda lignin (SL) have found industrial application in areas such as phenolic adhesives, nearly all of them are still burnt for energy at their production sites. Bark and many other parts of trees [4,5,6] are rich in tannins and are often exploited for energy and mulch for improving soil structure. However, tannins extracted from these residues have the potential for value-added applications in many areas. Commercially extracted tannins are mainly used for leather tanning and, to a much lesser extent, in adhesive resins. While their production volume is low compared with lignins, they still offer a viable option for functional coatings.

In terms of coatings, lignins and tannins have several attractive functional properties to offer. Their UV light absorption and antioxidant properties have prompted research into their use in broad-range sunscreens and cosmetic formulations [2,7,8]. They also possess antimicrobial properties [2,3,8,9,10,11,12,13,14,15,16,17], thus having the potential to be applied as coatings in high-hygiene environments such as hospitals. Further, due to their char-forming properties and ability to abstract fire-promoting free radicals, they could be modified and then substituted for effective but toxic halogenated flame retardants (FRs) [18,19,20]. They could also replace intumescent polyphosphate-based FRs in coatings and bulk materials at a time when phosphorus resources are prioritized for food production.

Tannins have proven antimicrobial activities [9,10,16,17] associated with their ability to interact with proteins in extracellular microbial enzymes and cell walls and membrane surfaces of microbes. They may also deprive microbes of iron, a crucial element for many of them. The exact mechanisms of the antimicrobial action of individual tannins are not clear. This is partly because tannins are typically poorly characterized and heterogeneous mixtures of different oligomers. In addition, the configurational differences between epimers or otherwise similar tannins can affect their antimicrobial properties [17], and if they are non-commercial, they may only be partly soluble or insoluble in water. With regard to lignins such as the fully or mostly water-insoluble kraft, soda, and organosolv lignin of high molar mass [3], they tend to show fairly low antimicrobial activity unless dissolved in organic media or converted to nanoparticles to increase their surface area. In some cases, it is possible to obtain low-molar-mass lignin fractions soluble in water at neutral pH that show notable antibacterial activity in tests based on the diffusion of bioactive compounds [3,11]. The mechanisms of antibacterial action may be similar to those of tannins and dependent on the type and content of functional groups on the aromatic rings (phenolic hydroxyl and methoxyl groups) and side chains and the type of condensed structures between lignin phenylpropane units [2,3,14].

Both lignins and tannins [20,21] have certain drawbacks that limit their efficacy and scope of application in functional coatings. Dry, high-molar-mass technical lignins, mostly insoluble in water at acidic and neutral pH levels, tend to be large, irregular, and mostly microsized particles with a low surface area. As their contact area with microbes is low, they do not possess significant antimicrobial properties unless the testing is conducted in organic solvents to dissolve them [13,22]. However, the antimicrobial properties exhibited in organic solvents are not pertinent to any real-life exposure scenario. Large particles are also poorly suited for application as water-based functional coatings, as they do not form stable dispersions in water as required for spray- and dip-coating applications. Moreover, the coatings of such particles applied through other water-based methods typically show poor adhesion to the substrate and low cohesion, arising from the insufficient contact of the particles with the substrate and with each other. Moreover, the use of toxic organic solvents such as dioxane to facilitate the application of lignin coatings [13,23] is inconsistent with the current emphasis on green and safe industrial practices and products. By contrast, commercial tannins are water-soluble at room temperature, making them suitable only for coatings for which water resistance is not required, such as single-use hygienic garments or curtains. Furthermore, while lignins and tannins can impart a certain degree of flame retardancy [18,19], it tends to be less than what is achieved with commercial FRs, unless they are first modified by introducing nitrogen and/or phosphorus into their structures [18,20,24,25,26,27]. The main mechanisms of the FR action of polyphenols are related to the formation of a thermally insulating char layer in the condensed phase and the abstraction of free radicals in the gas phase [27]. The char also protects the underlying material from oxygen and flammable combustible products. Phosphorus-containing moieties of modified polyphenols help dehydrate the polyphenols, thus increasing the char yield, while nitrogen-based functional groups produce gasses that enhance the intumescence of the char layer.

Many of the above-mentioned issues hampering the use of lignins and tannins for functional coatings can be solved by converting them to nanoparticles (NPs) and small (ca. 1001–2000 nm) microparticles (MPs) of high surface area. Water-insoluble lignins and tannins that have not been chemically modified can be readily converted into aqueous NP dispersions through a solvent-exchange process, whereby tannin or lignin is first dissolved in a non-toxic and easily recyclable organic solvent (e.g., acetone) or a mixture of organic solvent and antisolvent, and the solution is then combined with an antisolvent to bring about NP formation. In most cases, water is the most practical choice for the antisolvent. The resulting lignin NP (LNP) or tannin NP (TNP) dispersions can be used for coating application as such, or the organic solvent can first be evaporated off and recycled in the process. If beneficial to the coating application method, the dispersions can be concentrated via high-speed centrifugation, lyophilization, or spray-drying.

Commercial condensed tannins (e.g., mimosa and quebracho tannins) and hydrolyzable tannins (e.g., chestnut, valonea, and tara tannins) are water-soluble at room temperature and therefore unsuitable for TNP synthesis as such if water is used as the antisolvent. Such tannins have been combined with other materials such as polyvinyl alcohol [28] and metal ions [29] to prepare tannin-based hybrid NPs that are stable in water. However, non-commercial alkali-extracted spruce bark tannin [4] is insoluble in water at room temperature and thus potentially able to be used for TNP synthesis as such.

The goals of the present work were to (1) synthesize NPs from water-insoluble tannin and lignin and characterize them; (2) elucidate the antibacterial properties of thin TNP and LNP spray coatings; and (3) introduce nitrogen into the tannin and lignin structures, convert the resulting N-modified tannin and lignins into NPs and small microparticles (MPs), and ascertain the FR properties of thick coatings fabricated from these particles. These types of coatings could find application in hospitals and other areas of high-hygiene requirements or in insulation materials or construction elements such as building claddings for which fire safety is important.

In terms of novelty, there is no published research on (1) TNPs prepared from tannin only (i.e., without metals, polyvinyl alcohol, or other major components besides tannin), (2) the synthesis of TNPs, LNPs, and small lignin MPs from nitrogen-modified tannin and lignin, and (3) the preparation of functional coatings from these particles.

## 2. Material and Methods

### 2.1. Chemicals, Tannins, and Lignins

Chemicals and solvents were of analytical grade and purchased from Sigma-Aldrich, Steinheim, Germany. The tannins, T100 and T160, were extracted from industrial spruce (*Picea abies*) bark via alkali extraction at 100 °C with 15% NaOH for 90 min (T100) or at 160 °C with 24% NaOH for 90 min (T160). The extracted tannins in the black liquor were then recovered through acid precipitation. More details on the extraction and recovery of the tannins can be found in Borrega et al. [4]. Softwood kraft lignin (SW KL) and hardwood (eucalyptus) kraft lignin (HW KL) were of industrial origin, while SW and HW CatLignins were prepared from industrial kraft black liquors using heat [30]. Wheat (*Triticum aestivum*) straw soda lignin was Protobind^TM^ 1000, GreenValue S.A., Granit, Switzerland. Beech (*Fagus sylvatica*) organosolv lignin (beech OSL) was obtained from Fraunhofer CBP, Leuna, Germany. Industrial bagasse, bamboo, and eucalyptus OSLs of unknown species were provided for free by Guangzhou Yinnovator Bio-technology Co., Ltd., Guangzhou, China. Ethanol-/water-based solvent fractionation was performed on SW-KL according to a published method [31], providing an insoluble (SW KL-HighMW) fraction at 80% ethanol content, and the fractions precipitated (SW KL-MediumMW) or remained soluble (SW KL-LowMW) at 50% ethanol content.

### 2.2. Characterization of Tannin and Lignins

The hydroxyl and carboxyl contents of tannins and lignins were determined from freshly phosphitylated tannins using ^31^P NMR on a Bruker 500 MHz NMR spectrometer at room temperature using a previously published method [32]. Molar mass measurements of tannins, dissolved in 0.1 M NaOH and filtered (0.45 μm), were performed by size exclusion chromatography (SEC) on PSS MCX 1000 and 100,000 Å columns using 0.1 M NaOH as the eluent (pH 13, 0.5 mL/min, T = 25 °C). The elution curves were detected using a Waters 2998 Photodiode Array detector at 280 nm. The weight (M_w_)- and number (M_n_)-average molar masses were calculated against polystyrene sulfonate standards (eight standards with a range of 3420−148,500 g/mol) using Waters Empower 3 (Milford, MA, USA) software. C, H, N, and S were determined for unmodified and nitrogen-modified tannins and lignins [33]. Methoxyl groups were determined using a published method [34] based on the gas chromatography (GC) determination of methyl iodide, formed from lignin methoxyl groups via the treatment of hydroiodic acid, with some modifications.

### 2.3. Nitrogen Modification of Tannin and Lignin

Nitrogen-modified T160 tannin and HW KL were prepared through treatment with formaldehyde and urea using a previously published method [25].

### 2.4. Synthesis of NPs and MPs

#### 2.4.1. Solvent Selection

In most cases, aqueous acetone containing 80% acetone by weight was used to dissolve the tannins and lignins for NP and MP synthesis. However, for the four tannins and lignins selected for coating experiments, a solubility study was performed to determine the optimal acetone concentration for each of them (Figure S1, Supplementary Materials). To estimate solubility, 0.1% solutions were prepared and then centrifuged at a high speed (14,000 rpm) for 20 min. The insoluble compounds were determined gravimetrically after drying in an oven at 105 °C for 90 min. The acetone concentration giving the least amount of insoluble residue was then selected for polyphenol dissolution (determined gravimetrically from an oven-dried sample).

#### 2.4.2. Solvent Exchange

Tannin or lignin was dissolved in aqueous acetone (acetone content was either 80% or selected as described above). The solution was then rapidly poured into a vigorously stirred beaker of water whereby NP (unmodified tannin and lignins, nitrogen-modified tannin) or mixed NP/MP dispersions (nitrogen-modified lignin) were formed at a tannin or lignin concentration of 0.8%. The acetone and some of the water were evaporated by stirring the dispersions overnight. As a result, the particle concentrations increased to ca. 1.3%.

### 2.5. Characterization of NPs and MPs

#### 2.5.1. Determination of Particle Size

The size distributions of lignin and tannin particles were determined via dynamic light scattering (DLS) on a Malvern Nano ZS90 Zetasizer (Malvern, UK) fitted with a 633 nm He-Ne laser. The measurements were performed at 173° in the backscattering mode at a water viscosity of 0.89 cP and reflective index of 1.33 at 25 °C. Approximately 15 to 20 subruns, adjusted automatically by equipment, were performed for each measurement. Three to five parallel measurements per sample were performed. The particle sizes are reported as intensity-based (M_i_), number-based (M_n_), and volume-based (M_v_) hydrodynamic diameters.

#### 2.5.2. Determination of ζ-Potential

The above-mentioned zetasizer was also utilized for the ζ-potential determinations. Z-potential data were obtained from electrophoretic mobility data by applying the Smoluchowski model. The zetasizer was used in the backscattering (173 °C) mode, with water set as dispersant at a viscosity of 0.89 cP and an RI (refractive index) of 1.330, and a lignin RI of 1.610. For the measurements, the samples were diluted to 0.09% (*w*/*v*), and the pH in all the measurements was maintained at 7 using a 10 mM NaCl solution. One milliliter of the sample was placed in a cuvette and kept at equilibrium at 25 °C for 60 s prior to the measurement. The measurements were performed using a DTS1070 dip-cell probe and repeated five times for each sample to check reproducibility. A voltage of ≤5 V was applied.

#### 2.5.3. Determination of Particle Morphology

The morphologies of dry lignin and tannin particles, obtained by freeze-drying the particle dispersions, were determined via scanning electron microscopy (SEM) on a Zeiss Merlin FE-SEM instrument. For improving secondary electron emission and thermal conduction, dried particles were coated with an ultra-thin (2 nm thick) electrically conducting Au/Pt layer. For SEM analysis, electron high tension (EHT) was applied at 2–3 mV.

### 2.6. Antibacterial Properties

#### 2.6.1. Determination of Minimum Inhibitory Concentrations

The minimum inhibitory concentration (MIC) tests of NPs were performed according to a standard method [35]. The test bacteria used were *Staphylococcus aureus* (*S. aureus*) VTT E-70045 (VTT Culture Collection, VTT Technical Research Centre of Finland Ltd, Espoo, Finland) and *Escherichia coli* (*E. coli*) VTT E-94564.

#### 2.6.2. Antibacterial Efficacy of Nanocoatings

The antibacterial efficacy of coated surfaces was tested using the test setup from ISO standard 22196:2011(E) “Measurement of antibacterial activity on plastics and other non-porous surfaces” in which o/n cultured methicillin-resistant *Staphylococcus aureus* (MRSA; VTT E-183582; 2.4 × 10^8^ bacteria) in nutrient broth (0.4 mL) was used to contaminate a 5 cm × 5 cm sample and the inoculum was covered with sterile 4 cm × 4 cm plastic. After 24 h exposure time at room temperature, the bacteria were rinsed off with 10 mL peptone saline. The diluted samples were then cultured on plate count agar and incubated at 37 °C for 24 h. The survival of bacteria on coated surfaces was compared with the number of bacteria on the reference sample, which was a 0.1 mm thick uncoated Whatman 1 filter paper.

### 2.7. Coating Preparation

#### 2.7.1. Coatings for Antibacterial Testing

A ca. 1.3% TNP dispersion, prepared from T160 as described earlier, was spray-coated onto Whatman 1 cellulose filter paper with a diameter of 15 cm and a thickness of 0.1 mm using a spray bottle with a manual pump. The cellulose paper was conditioned to equilibrium moisture content (EMC) at 23% rh and 25 °C before and after coating application before it was weighed to calculate coating coverage.

#### 2.7.2. Coatings for Microscale Combustion Calorimetry (MCC) Testing

Particle dispersions were prepared from unmodified and nitrogen-modified tannin and lignin as described earlier and then concentrated via high-speed centrifugation (14,000 rpm). The concentrates were then brush-coated onto preweighed pieces of cellulose (1 mm thick sheets of bleached HW kraft pulp, ca. 929 g/m^2^), conditioned to EMC at 23% rh and 25 °C before weighing. The coated sheets were dried at 40 °C and then conditioned to EMC at the above conditions before being weighed to determine coating coverage.

### 2.8. Microscale Combustion Calorimetry

Microscale combustion calorimetry (MCC) is an experimental method for measuring the heat release rate of a small sample (ca. 1–10 mg) as a function of temperature [36,37]. It reveals how much combustible gases evolve and how much energy is released in the pyrolysis of the specimen tested. In this work, the peak heat release rate (PHRR), the temperature at PHRR (T_PHRR_), and the total heat released (THR) of lignin- and tannin-coated cellulose samples were determined using a Model-MCC-2 Govmark Microscale Combustion Calorimeter (New York, NY, USA) in a nitrogen atmosphere at a heating rate of 1.0 K/s. The char yield, defined as the percentage of solid residue remaining after MCC relative to the initial weight of the test specimen, was determined gravimetrically. Two replicate tests were performed for each material.

## 3. Results and Discussion

### 3.1. Characterization of Tannins and Lignins

The unmodified tannins T100 and T160 contain ca. 55% tannin, while T160 additionally contains ca. 21% lignin, the balance being made up of carbohydrates, protein, and inorganic material quantified as ash [4]. Because of the higher solubility of T160 in the solvent used for TNP synthesis (86.4% for T160 vs. 73.2% for T100 in 80% acetone), T160 was selected over T100 for coating preparation.

The lignins showed large differences in their functional group contents (Table 1) in line with their botanical origins, production conditions, and/or fractionation process. *p*-Hydroxyphenyl units (H-units) were found in significant quantities in grass-type lignins (bamboo, bagasse, and wheat straw) and kraft lignins that had undergone partial demethoxylation (CatLignins). Phenolic hydroxyl contents were highest for kraft lignins (KLs) and in particular for the CatLignins rich in catechol units resulting from the partial demethylation of lignin. The extensive demethoxylation and demethylation of the CatLignins were also reflected in their low methoxyl contents. The fractions obtained through the solvent fractionation of KL significantly differed in terms of molar mass and phenolic hydroxyl content. These properties reflect the extent of depolymerization undergone by these lignin fractions, a lower molar mass being associated with a higher phenolic hydroxyl content arising from the cleavage of mainly b-aryl ether bonds.

The elemental analysis of the nitrogen-modified tannins and lignins and their corresponding starting materials, determined in an earlier investigation [24], showed that the modification using the Mannich reaction (and possibly the Schiff base reaction for the lignin) introduced over 10% nitrogen to their structure. As these polyphenols were only partially soluble in NMR solvents and reagents, they were not characterized using NMR.

### 3.2. Characterization of TNPs and LNPs

**Figure 1:**
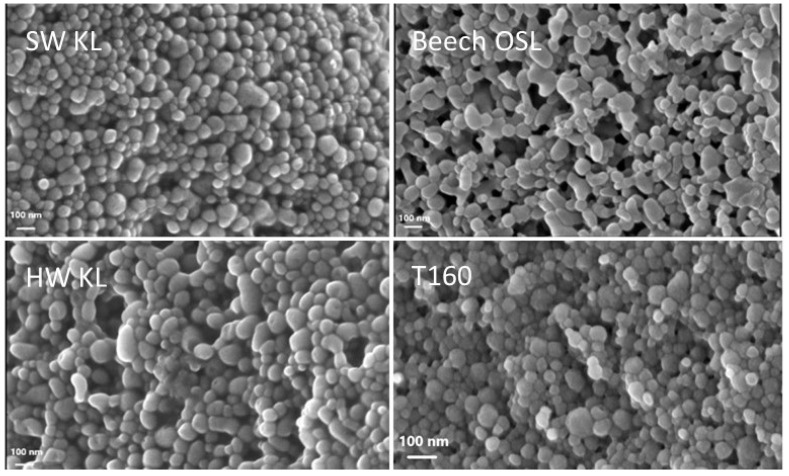
SEM images of selected LNPs and TNPs.

The spruce bark tannins, T100 and T160, gave spherical TNPs of similar sizes and ζ-potentials (Table 2) despite their somewhat different molar masses and hydroxyl group contents (Table 1) and the presence of lignin in T160 along with tannin [4]. The lignins formed LNPs of different sizes and shapes: SW LNPs were spherical, while HW and grass-type LNPs were elongated or had a mixture of spherical and elongated particles. The SEM images of some of the NPs are illustrated in Figure 1. Based on a comparison of the lignin unit type distribution (*p*-hydroxyphenyl, guaiacyl, syringyl, and catechol; Table 1) of the lignins with their particle shapes, it seems that the presence of syringyl units is required to produce elongated particles, and, in their absence, spherical shapes are obtained. The surface charges of LNPs fell over a wide range with their ζ-potentials ranging from −35 mV to −51 mV. The LNPs produced from N-modified HW KL were significantly larger than those made from HW KL, but their ζ-potentials were similar.

As discussed earlier, the unmodified lignins were very heterogeneous in terms of functional group distribution and molar mass (Table 1). To establish high correlations between the properties of lignins and LNPs, it was necessary to split the lignins into three structurally more homogeneous groups that were analyzed separately (Table 3): SW KLs (*n* = 4); organosolv and soda HW and grass lignins (*n* = 5); and SW and HW kraft and CatLignins (*n* = 4).

For the SW KLs, a low molar mass, particularly the M_n_ value, was a good predictor of low LNP ζ-potential. A low LNP M_n_ in turn was highly correlated with a low lignin M_n_. This can be rationalized as the smaller LNPs having a larger specific surface area and thus a larger contribution to the surface charge (ζ-potential) than larger LNPs with a smaller specific surface area. Moreover, a low lignin M_n_ was negatively correlated with a higher content of hydrophilic (hydroxyl and carboxyl) groups because, via the cleavage of alkyl–aryl ether bonds, lignin depolymerization increased the content of phenolic hydroxyls, the most prominent hydrophilic functional group. A higher content of these functional groups at the LNP surface likely accounts for the lower ζ-potential of the smaller LNPs as defined by their M_n_ value.

Regarding the second group that comprised organosolv and soda lignins, the relationship between molar mass (M_w_ in this case) and LNP size (M_n_) was reversed compared with the first group of lignins: higher molar mass resulted in smaller LNPs, while LNP size (as defined by M_i_ and M_v_ values in particular) was highly correlated with more negative ζ-potential, the smaller particles bearing a more negative surface charge. In addition, a higher hydrophilic group content of lignin predicted a higher M_n_ value, not a lower one as in the case of the SW KLs.

In the third group that involved SW and HW KLs and CatLignins, all three LNP size parameters (M_i_, M_v_, M_n_) were strongly intercorrelated with a smaller size correlating with a more negative ζ-potential. While there were no significant correlations between lignin and LNP properties across the entire group, it should be noted that the SW and HW CatLignins possessed more hydrophilic functional groups and had more negative ζ-potential than the corresponding SW and HW KLs. The two CatLignins and bagasse OSL gave the smallest particles overall and also had the lowest methoxyl group content, suggesting that the combination of very low methoxyl content and high hydrophilic functional group content might be conducive to particularly small particle size.

Across all the unmodified lignins, significant correlations were hard to find due to their structural heterogeneity, but a smaller LNP M_n_ value was in general a good predictor of lower ζ-potential at an r^2^ value of 0.63.

### 3.3. Antibacterial Properties of Nanoparticles and Nanocoatings

**Figure 2:**
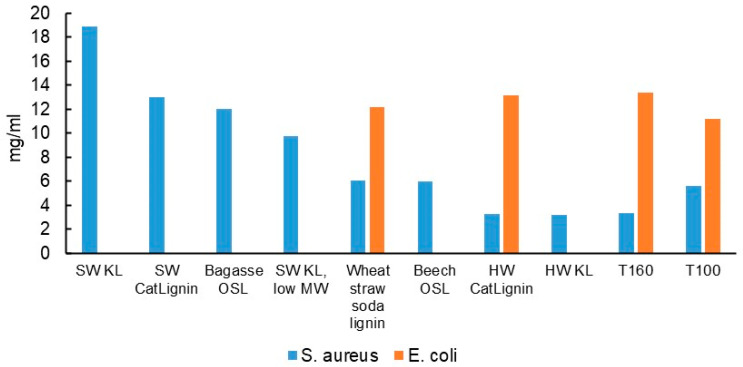
Minimum inhibitory concentration (MIC) of tannins and lignins for *S. aureus* and *E. coli*. The other lignins listed in Table 1 had *S. aureus* MICs of >20 mg/mL. *E. coli* MICs were determined only for NPs with *S. aureus* MICs of <10 mg/mL. For those samples, a missing bar indicates a MIC of >20 mg/mL.

The minimum inhibitory concentrations (MICs) of all the TNPs and LNPs listed in Table 1 for *S. aureus* were determined up to the limit of 20 mg/mL (Figure 2). For those NPs with a MIC of 10 mg/mL or lower for *S. aureus*, their MIC for *E. coli* was also determined. The results show that TNPs, HW KL LNPs, and HW CatLignin LNPs were the most effective NPs against *S. aureus*. With the exception of HW KL, they also inhibited *E. coli*, albeit to a lesser degree than *S. aureus*. The MIC values could not be predicted from the NP properties in Table 2. However, the MICs for the most effective LNPs against *S. aureus* declined with a decrease in lignin M_w_, low-MW SW KL being an outlier. Further evidence for the possible significant effect of molar mass on antibacterial activity was provided by the comparison of eucalyptus and beech OSLs. Despite these lignins having nearly identical phenolic hydroxyl and methoxyl contents, LNPs of beech OSL were among the most effective ones (MIC 6 mg/mL), while those of eucalyptus OSL were ineffective (MIC > 20 mg/mL). It is also observed that only the lowest MW fraction of SW KL gave a measurable MIC at 9.8 mg/mL, its mid- and high-MW fractions being ineffective. As for the two tannins, TNPs of T160 (M_w_ 2900) were more effective than those of T100 (M_w_ 4000). These results suggest that while a relatively low molar mass of lignin or tannin is a central feature of corresponding antibacterially efficacious LNPs and TNPs, there are also other factors at play that could not be elucidated in this investigation.

**Figure 3:**
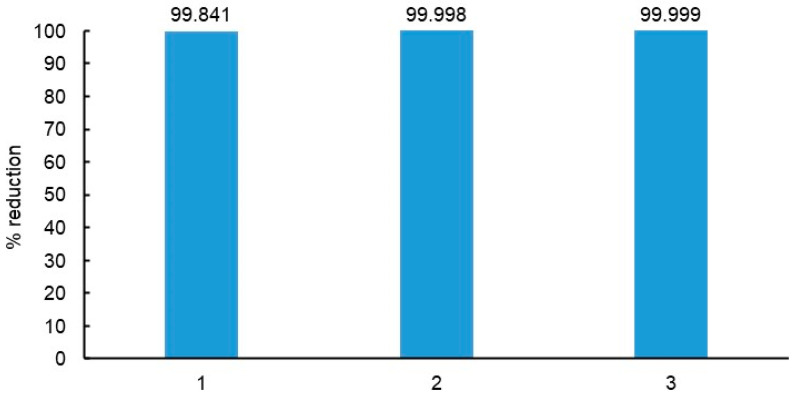
MRSA reduction of TNP-coated (T160) cellulose relative to uncoated cellulose. Coating 1 was applied via dipping and the replicate coatings 2 and 3 via spraying. Coating coverage was ca. 5 g/m^2^, and starting MRSA level was 2.4 × 10^8^.

The antibacterial properties of T160-TNPs that showed efficacy against both test bacteria in the MIC test were further investigated as antibacterial coatings on cellulose (Figure 3). Compared with uncoated cellulose that showed essentially no growth or decrease in bacteria applied onto their surface, the coating applied through dipping was bacteriostatic, reducing MRSA by 99.8%, while the two coatings applied via spraying were bactericidal with an MRSA reduction of >99.99%.

### 3.4. FR Properties of Nano/Microcoatings

To facilitate coating application via brushing, the respective particle dispersions, prepared using the optimal acetone concentration for each polyphenol (Figure S1, Supplementary Materials), were concentrated via high-speed centrifugation prior to use. For T160 and N-T160, the TNPs in the concentrate were of nearly similar size to those in the original dispersion, but their ζ-potential was notably lower (Table 4). It is also seen that the N-modification of T160 increased the particle size and reduced the ζ-potential. As for HW-KL, the LNPs in the supernatant accounted for only 10% of the total, which is reflected in the similarity of the particle sizes and ζ-potentials of the original dispersion and the concentrate. On a mass basis, most of the particles in the original N-HW KL and its concentrate were microsized—more than 8–10 times in size than those of HW KL—and had a considerably lower ζ-potential. The nanosized particles mostly ended up in the supernatant. In general, there seemed to be no clear relationship between the supernatant and concentrate yields and particle characteristics.

At first, TNPs and LNPs prepared from unmodified or N-modified tannin or lignin were brush-coated onto both sides of the pieces of cellulose sheet, and the coated pieces were analyzed via microscale combustion calorimetry (MCC). The results show clear improvements in the MCC parameters’ peak heat release rate (PHRR), total heat released (THR), and char residue compared to uncoated cellulose. It should be noted that these results cannot be used to compare the efficacy of different types of NPs because of the different coating coverages applied (10–89 g/m^2^ or ca. 1–10% wt.-% of the coated cellulose pieces). Since an earlier investigation [24] had established the superior FR properties of N-modified tannins and lignins compared with their unmodified counterparts, the focus of this study was on the coatings prepared from N-modified tannins and lignins. The MCC test results of NP-coated cellulose specimens are presented in Table 5 as PHRR, T_PHRR_, THR, and char yield values. The repeatability of the replicate tests was good, as can be seen in Table 5.

Compared with uncoated cellulose sheets, all the NP coatings studied improved the heat release performance. For T160 at 10 g/m^2^, the reduction in PHRR and THR were 7% and 6%, respectively. N-HW KL at 48 g/m^2^ decreased PHRR by 14% and THR by 5%. The most significant improvements were seen for N-T160 coatings. Layers of 61 and 89 g/m^2^ reduced PHRR by 32% and 39% and THR by 19% and 22%, respectively. However, these coatings brought T_PHRR_ to a lower value. No change in T_PHRR_ was seen for 10 g/m^2^ T160 and 48 g/m^2^ N-HW KL coatings. The char yield was slightly increased for 10 g/m^2^ T160 and 48 g/m^2^ N-HW KL. For N-T160 coatings, the char yield was almost doubled compared with the uncoated reference specimen.

**Figure 4:**
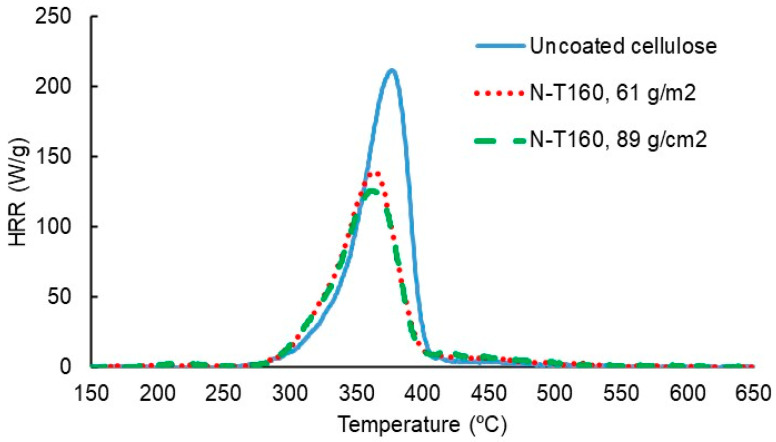
Heat release rate (HRR) in the MCC tests of uncoated cellulose (ref) and N-T160-coated cellulose. The result of test 1 is shown for each specimen.

Figure 4 illustrates the MCC test performance of uncoated cellulose sheets (ref) and two N-T160-coated cellulose sheets. The effect of N-T160 coating on heat release rate (HRR) compared with uncoated cellulose can be clearly seen. Due to the good repeatability, only the result of test 1 for each specimen is presented.

## 4. Conclusions

LNPs prepared from different technical lignins and TNPs of spruce tannin showed large differences in regard to their antibacterial efficacy against *S. aureus* bacteria. A common factor among the most effective LNPs such as those composed of HW kraft lignin was a relatively low molar mass of the lignin, but that alone did not guarantee high efficacy. TNPs showed good antibacterial activity both in suspension testing and especially as coatings on cellulose, reducing MRSA by more than 4 log units. The nanoparticles of nitrogen-modified spruce tannin and small microparticles of nitrogen-modified HW kraft lignin exhibited promising flame-retardant properties when applied as coatings on cellulose.

## Figures and Tables

**Table 1:** Functional group content and molar mass of unmodified tannins and lignins.

Tannin/ Lignin	Functional Group, mmol/g								Molar Mass, g/mol		
	Phenolic OH *					Aliphatic OH	COOH	OCH_3_	M_w_	M_n_	PDI
	H	G	C + S	Cat	Total						
T100					2.9	3.9	1.0	0.03	4000		3.5
T160					3.5	2.1	1.3	1.10	2900		2.8
SW KL	0.23	2.46	2.05	0.00	4.74	1.99	0.43	4.55	5010	2277	2.2
SW KL-LowMW	0.28	3.51	1.72	0.00	5.51	1.61	0.57	4.28	1590	1060	1.5
SW KL-MediumMW	0.25	2.20	1.90	0.00	4.35	1.37	0.49	4.07	3390	1994	1.7
SW KL-HighMW	0.21	1.82	1.83	0.00	3.86	2.00	0.31	3.61	9570	2991	3.2
SW CatLignin	0.70	1.20	2.40	1.20	5.50	0.90	0.70	2.19	3700	1700	2.2
Beech OSL	0.08	0.56	2.44	0.35	3.43	1.80	0.12	6.32	1890	943	2.0
Eucalypt OSL	0.10	0.38	2.59	0.28	3.35	0.62	0.19	6.33	4092	981	4.2
HW KL	0.20	0.49	3.40	0.50	4.59	1.30	0.30	5.60	2050	1170	1.8
HW CatLignin	1.17	0.67	3.05	1.87	6.76	0.29	0.83	2.75	2330	1370	1.7
Bamboo OSL	0.69	0.41	1.57	0.32	2.99	0.66	0.32	5.07	2927	809	3.6
Bagasse OSL	0.86	0.40	1.14	0.33	2.73	0.70	0.59	3.60	3611	806	4.5
Wheat straw SL	0.47	0.53	1.96	0.44	3.40	1.43	0.96	4.60	3300	2000	1.7

* H = *p*-hydroxyphenyl, G = guaiacyl, C + S= condensed + syringyl, Cat = catechol.

**Table 2:** Nanoparticle properties.

Polyphenol	Particle Size Distribution, nm			Predominant Particle Shape(s)	Z-Potential, mV
Tannins	M_i_	M_v_	M_n_		
Tannin T160	81	70	54	Spherical	−40.7
Tannin T100	99	152	52	Spherical	−38.4
N-T160	113	95	49	n/a	−36.2
Lignins	M_i_	M_v_	M_n_		
SW KL	137	170	95	Spherical	−39.4
SW KL-LowMW	179	580	42	Spherical	−61.3
SW KL-MediumMW	117	109	79	Spherical	−44.7
SW KL-HighMW	314	728	109	Spherical	−36.6
SW CatLignin	52	41	26	Spherical	−51.4
HW KL	143	185	99	Elongated and spherical	−43.7
N-HW KL	430	310	794	n/a	−42.3
HW CatLignin	106	93	59	Elongated and spherical	−48.9
Wheat straw soda	169	226	119	Elongated and spherical	−41.4
Bagasse OSL	105	92	64	Elongated and spherical	−41.8
Bamboo OSL	157	285	96	Elongated and spherical	−38.7
Beech OSL	289	680	106	Mostly elongated, some spherical	−34.6
Eucalyptus OSL	114	104	69	Elongated and spherical	−42.2

**Table 3:** Correlation matrices (r^2^) of properties of groups of lignins vs. their LNPs.

Group 1		SW KL Properties			SW KL LNP Properties			
		OH and COOH, mmol/g	M_w_, g/mol	M_n_, g/mol	M_i_, nm	M_v_, nm	M_n_, nm	Z-Potential, mV
SW KL properties (n = 4)	OH and COOH, mmol/g	1	0.42 (−)	0.58 (−)	0.10 (−)	0.00 (+)	0.51 (−)	0.53 (−)
	M_w_, g/mol	0.42 (−)	1	0.90 (+)	0.63 (+)	0.19 (+)	0.80 (+)	0.68 (+)
	M_n_, g/mol	0.58 (−)	0.90 (+)	1	0.32 (+)	0.02 (+)	0.97 (+)	0.91 (+)
SW KL LNP properties (n = 4)	M_i_, nm	0.10 (−)	0.63 (+)	0.32 (+)	1	0.79 (+)	0.19 (+)	0.09 (+)
	M_v_, nm	0.00 (+)	0.19 (+)	0.02 (+)	0.79 (+)	1	0.00 (−)	0.02 (−)
	M_n_, nm	0.51 (−)	0.80 (+)	0.97 (+)	0.19 (+)	0.00 (−)	1	0.98 (+)
	Z-potential, mV	0.53 (−)	0.68 (+)	0.91 (+)	0.09 (+)	0.02 (−)	0.98 (+)	1
Group 2		HW and grass lignin properties *			HW and grass LNP properties *			
		OH and COOH, mmol/g	M_w_, g/mol	M_n_, g/mol	M_i_, nm	M_v_, nm	M_n_, nm	Z-potential, mV
HW and grass lignin properties *	OH and COOH, mmol/g	1	0.22 (−)	0.63 (+)	0.41 (+)	0.27 (+)	0.69 (+)	0.10 (+)
	M_w_, g/mol	0.22 (−)	1	0.01 (+)	0.88 (−)	0.93 (−)	0.39 (−)	0.93 (−)
	M_n_, g/mol	0.63 (+)	0.01 (+)	1	0.00 (+)	0.01 (+)	0.45 (+)	0.07 (+)
HW and grass LNP properties *	M_i_, nm	0.41 (+)	0.88 (−)	0.00 (+)	1	0.98 (+)	0.45 (+)	0.87 (+)
	M_v_, nm	0.27 (+)	0.93 (−)	0.01 (+)	0.98 (+)	1	0.35 (+)	0.95 (+)
	M_n_, nm	0.69 (+)	0.39 (−)	0.45 (+)	0.45 (+)	0.35 (+)	1	0.23 (+)
	Z-potential, mV	0.10 (+)	0.93 (−)	0.07 (+)	0.87 (+)	0.95 (+)	0.23 (+)	1
Group 3		KL and CatLignin properties			KL and CatLignin LNP properties			
		OH and COOH, mmol/g	M_w_, g/mol	M_n_, g/mol	M_i_, nm	M_v_, nm	M_n_, nm	Z-potential, mV
KL and CatLignin properties (n = 4)	OH and COOH, mmol/g	1	0.03 (+)	0.06 (+)	0.13 (−)	0.30 (+)	0.22 (−)	0.13 (−)
	M_w_, g/mol	0.03 (+)	1	0.98 (+)	0.01 (−)	0.00 (−)	0.00 (+)	0.17 (+)
	M_n_, g/mol	0.06 (+)	0.98 (+)	1	0.00 (−)	0.00 (+)	0.00 (+)	0.21 (+)
KL and CatLignin LNP properties (n = 4)	M_i_, nm	0.13 (−)	0.01 (−)	0.00 (−)	1	0.94 (+)	0.98 (+)	0.75 (+)
	M_v_, nm	0.82 (+)	0.00 (−)	0.00 (+)	0.94 (+)	1	0.99 (+)	0.82 (+)
	M_n_, nm	0.22 (−)	0.00 (+)	0.00 (+)	0.98 (+)	0.99 (+)	1	0.83 (+)
	Z-potential, mV	0.13 (−)	0.17 (+)	0.21 (+)	0.75 (+)	0.30 (+)	0.83 (+)	1

* HW kraft lignin excluded.

**Table 4:** Properties of original nano-/microparticles and their centrifugation fractions used for coatings.

Sample	Size (M_i_), nm			Z-Potential, mV			S/C ^1^ Yield Ratio
	Original	Supernatant	Concentrate	Original	Supernatant	Concentrate	
T160	126 ± 1	80 ± 0	133 ± 1	−24.8 ± 0.4	−20.7 ± 0.6	−35.3 ± 1.1	22/78
N-T160	214 ± 2	128 ± 1	222 ± 1	−34.0 ± 1.5	−35.5 ± 1.6	−39.7 ± 1.1	48/52
HW KL	176 ± 1	103 ± 1	199 ± 4	−44.3 ± 0.5	−43.0 ± 1.2	−45.8 ± 1.3	10/90
N-HW KL ^2^	2054 ± 83	325 ± 14	1590 ± 167	−52.7 ± 1.0	−48.9 ± 1.2	−48.5 ± 1.9	22/78

^1^ Supernatant/concentrate; ^2^ Size values >1000 nm have reduced reliability in zetasizer measurements.

**Table 5:** FR properties of NP- and MP-coated cellulose by MCC.

Coating	PHRR, W/g	T_PHRR_, °C	THR, J/g	Char Yield, %
Uncoated cellulose sheet (ref)	207 ± 5	378 ± 2	10200 ± 200	11.5 ± 0.8
T160, 10 g/m^2^	192 ± 2	380 ± 0	9630 ± 60	12.7 ± 0.5
N-HW KL, 48 g/m^2^	177 ± 1	377 ± 2	9700 ± 250	14.6 ± 0.9
N-T160, 61 g/m^2^	141 ± 2	364 ± 1	8290 ± 40	18.4 ± 0.6
N-T160, 89 g/m^2^	127 ± 1	362 ± 0	7960 ± 10	19.2 ± 0.2

## Data Availability

Data will be made available upon request.

## Supplementary Materials

The following supporting information can be downloaded at: https://www.mdpi.com/article/10.3390/polym15040992/s1. Figure S1: Solubility of tannins and lignins in aqueous acetone. T160 = spruce tannin alkali-extracted at 160 °C, N-T160 = nitrogen-modified T160, HW KL = hardwood kraft lignin, N-HW KL= nitrogen-modified HW KL.
