# Supplementary material for: Antimicrobial and Flame-Retardant Coatings Prepared from Nano- and Microparticles of Unmodified and Nitrogen-Modified Polyphenols
Source: Polymers (Basel). 2023 Feb 16;15(4):992. doi: 10.3390/polym15040992 (PMC9958896; doi:10.3390/polym15040992)
Supplement: Supplementary file 1 [file polymers-15-00992-s001.zip › polymers-2202641-supplementary.pdf]

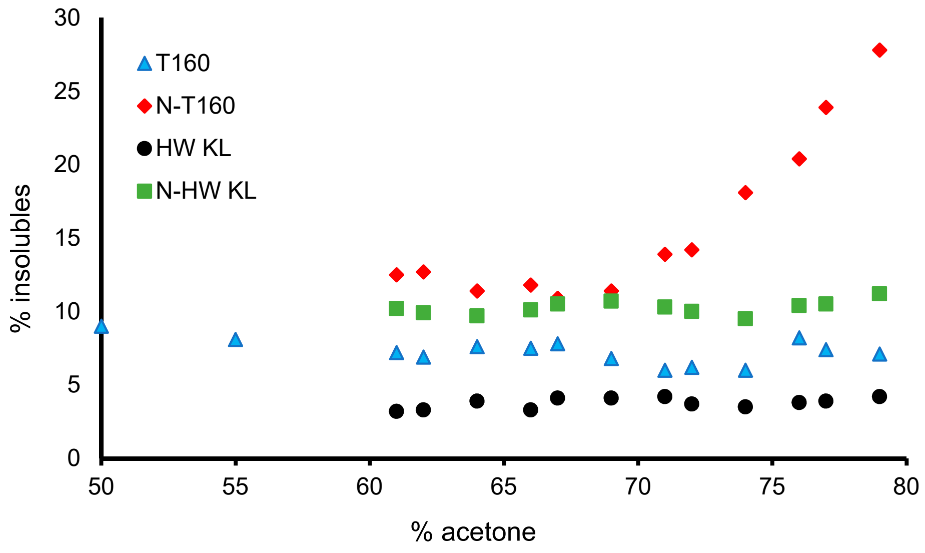

Figure S1. Solubility of tannins and lignins in aqueous acetone. T160 = spruce tannin alkali-extracted at 160°C, N-T160 = nitrogen-modified T160, HW KL = hardwood kraft lignin, N-HW KL= nitrogen-modified HW KL.
